# Effects of V-N Microalloying on Low-Cycle Fatigue Property in the Welded Joints of Constructional Steel

**DOI:** 10.3390/ma16175860

**Published:** 2023-08-27

**Authors:** Kaiyu Cui, Haifeng Yang, Zhengrong Li, Guodong Wang, Hongyun Zhao, Yuxuan Li

**Affiliations:** 1 State Key Laboratory of Advanced Welding and Joining, Harbin Institute of Technology, Harbin 150001, China; yjycuiky@pzhsteel.com.cn (K.C.); hf_yang@hit.edu.cn (H.Y.); wanggd@neu.edu.cn (G.W.); 2 State Key Laboratory of Vanadium and Titanium Resources Comprehensive Utilization, Pangang Group, Panzhihua 617000, China; yjylzr@pzhsteel.com.cn; 3 Shandong Provincial Key Laboratory of Special Welding Technology, Harbin Institute of Technology (Weihai), Weihai 264209, China; liyuxuanhit@163.com; 4 School of Materials Science and Engineering, Harbin Institute of Technology (Weihai), Weihai 264209, China

**Keywords:** vanadium, nitrogen, constructional steel, welded joints, low-cycle fatigue, seismic resistance

## Abstract

Low-cycle fatigue testing was carried out for the welded joints of constructional steels containing 0% V + 0.0021% N and 0.10% V + 0.0078% N, and the effects of V-N microalloying on the low-cycle fatigue property of the welded joints were investigated. The results showed that when the total strain amplitudes were 1.2%, 1.4% and 1.6%, the mean low-cycle fatigue lives of the welded joints of steel containing 0.10% V + 0.0078% N were 5050, 2372 and 1535 cycles, respectively, which were significantly higher than those of the welded joints of steel containing 0% V + 0.0021% N; however, when the total strain amplitudes increased to 1.8% and 2.0%, the mean low-cycle fatigue lives of the welded joints of steel containing 0.10% V + 0.0078% N were 575 and 367 cycles, respectively, which were gradually lower than those of the welded joints of steel containing 0% V + 0.0021% N. The reasons causing the difference of low-cycle fatigue life were explained by the dislocation structure and precipitates in the welding heat-affected zone, plastic strain energy density of the welded joints, and fatigue fracture morphology. When the low-cycle fatigue life is between 100 and 200 cycles, the cyclic toughness of the welded joint of steel containing 0.10% V + 0.0078% N is between 57.48 and 78.22 J/cm^3^, which is higher than that of the welded joint of steel containing 0% V + 0.0021% N, indicating that the welded joint of steel containing 0.10% V + 0.0078% N is able to absorb more energy in a seismic condition, therefore possessing better seismic resistance.

## 1. Introduction

Comparing with a traditional construction structure, the application of high-strength steel can significantly increase strength and decrease weight, reduce construction difficulty and improve the material recycling rate; therefore, steel structure construction is developing quite rapidly all over the world [1,2,3]. The welding of constructional steel is one of the main connection methods for steel structure construction, considering the microstructural transformation in the weld joint (WJ): especially in the heat-affected zone (HAZ), a brittle or soft zone usually occurs. Zhiqiang Zhang et al. [4] investigated the influences of N_2_ content in the shielding gas on the microstructure and impact toughness of the WJ of duplex stainless steel, and they found that the increase in N_2_ content significantly facilitated austenite formation in the WJ and increased the Cr_2_N precipitates content and dislocation density in the HAZ, thereby leading to the toughness of the WJ increasing first and then decreasing, and the HAZ had the lowest toughness. Fu et al. [5] investigated the hydrogen-induced stress corrosion cracking behavior of the WJ of SUS301L-MT stainless steel and found that the base metal (BM) and HAZ exhibited higher plasticity loss than that of the weld metal (WM) but almost same strength loss, and hydrogen embrittlement cracks both in BM and HAZ initiated along the interface of α′ martensites and austenite, while they initiated along the interface of δ-ferrite and austenite in the WM. Thus, the safety and service performance of the steel structural construction not only depends on the property of steel, but it also greatly depends on the property of the WJ. During earthquakes, the cyclic cycles of seismic waves are limited, and steel structure construction experiences low-cycle fatigue (LCF) under cyclic seismic load [6,7,8,9]. Therefore, in order to achieve excellent seismic resistance, not only the BM but also the WJ of constructional steel should possess excellent LCF property.

Some investigations on the framework for probabilistic fatigue curves prediction and the probabilistic fatigue life prediction model of alloys have been carried out [10,11]. Moreover, many investigations on the LCF property of the WJ of high-strength steel have been carried out, and it is generally recognized that microstructural transformation will lead to a change in the LCF property in the WJ. Wei Song et al. [12] carried out LCF testing for the BM and undermatched welds of 10CrNi3MoV steel, and they found that larger acicular ferrite and granular carbides in the base material led to transgranular cracking easily; according to the evaluation of plastic strain energy density and total strain energy density, the fatigue resistance of the undermatched weld was better than that of the BM. Shuo Weng et al. [13] investigated the mechanical property and microstructural transformation of the BM and WJ of NiCrMoV steel under the LCF condition, and they found that cyclic softening took place in the BM and WJ under various strain amplitudes. The decrease in dislocation density in the WJ was mainly attributed to the dislocation pile-ups along the grain boundaries and the formation of subgrains, which led to the decrease in the WJ strength. Tomasz Ślęzak [14] carried out LCF testing for the BM and two types of WJ, and they found that the LCF property of the square joints was better than that of the single-V joints, which was attributed to more favorable hardness distribution and fewer welding defects. Yaqi Suo et al. [15] applied low cycle load to the steel structures produced by the welding of Q235 and Q345, and they found that cyclic softening took place in the WJ, and the cyclic stress amplitude and energy dissipation capacity decreased gradually with the increase in cyclic cycles, while the fatigue damage accumulation rate in the WJ increased with the increase in cyclic stress amplitude. J. W. Sowards et al. [16] carried out LCF testing for the BM and WJ of corrosion-resistant high-strength low alloy steel, and they found that the formation of martensite in the WJ caused by rapid cooling inherent to laser welding resulted in the significantly higher LCF strength of the WJ than that of the BM. Moreover, the LCF life of the WJ was shorter than that of the BM when the plastic strain was high, but it was close to that of the BM at lower plastic strain. Wenyuan Zhang et al. [17] carried out LCF testing with a uniaxial load or axial–torsional load for the WJ of Q355B, and they found that the strain of the BM and WJ was close when the loading range was small, but the large loading range made the WJ more likely to develop plastic strain, which led to the fatigue crack mainly taking place at the WJ. J. Veerababu et al. [18] carried out LCF testing for the BM and WJ of grade 92 steel, and they found that cyclic softening took place in the BM and WJ, but the cyclic stress of the WJ was lower than that of the BM. Moreover, the LCF life of the WJ was shorter than that of the BM at low strain amplitude, while the LCF life of the WJ was comparable to that of the BM at high strain amplitude.

Considering that the V element is a strong carbonitride-forming element, a certain content of V and N elements are usually added into high-strength steel to obtain precipitates containing V inside grains and at grain boundaries to realize precipitation strengthening and refinement strengthening, thereby improving the properties of steel such as strength, plasticity and toughness [19,20,21,22], and some investigations on the effects of precipitates containing V on the LCF property of steel have been carried out [23,24]. During welding, the dissolution, growth and precipitation of V carbonitride in the weld thermal cycle will affect the microstructural transformation of the WJ [25,26,27,28], thereby affecting the LCF property of the WJ. However, the effects of V-N microalloying on the LCF property in the WJ of constructional steel were investigated rarely. Therefore, the present paper investigated the LCF property in the WJs of steels containing 0% V + 0.0021% N and 0.10% V + 0.0078% N. The steel containing 0.10% V + 0.0078% N possesses high strength, good plasticity and a low yield ratio to satisfy the seismic resistance requirement of the steel structure, and it has good weathering resistance to reduce the risk of failure due to corrosion, therefore having great potential for application in the field of steel structure construction. The present paper carried out shielded metal arc welding for constructional steels containing 0% V + 0.0021% N and 0.10% V + 0.0078% N, and LCF testing for the welded joints (WJs), to investigate the effects of V-N microalloying on the LCF property and seismic resistance of the WJs of constructional steel through LCF life, cyclic stress response curves, hysteresis loops, fatigue fracture morphology and cyclic toughness.

## 2. Materials and Methods

### 2.1. Materials

Experimental materials were smelted by a VAR-150 consumable electrode vacuum furnace (Baotai Equipment Technology Co. Ltd., Baoji, China), and the 70 mm billets were rolled into a 6 mm thick steel plate with an 860 °C finish temperature and 650 °C roiling temperature. The chemical composition and carbon equivalent values of the tested steels are shown in Table 1. In order to reduce the risk of failure due to corrosion, certain contents of Cu, Ni, and Cr were added into both tested steels to increase the weathering resistance. The 0 V tested steel contained 0% V and 0.0021% N, while the 10V8N steel contained 0.10% V and 0.0078% N. According to the calculation equation of carbon equivalent value (CE) suggested by the International Institute of Welding (IIW) (Equation (1)), the values of CE of tested steels were high to reduce the weldability.

(1) $$\mathrm{CE}=C+\frac{Mn}{6}+\frac{Cr+Mo+V}{5}+\frac{Ni+Cu}{15}$$

### 2.2. Methods

#### 2.2.1. Shielded Metal Arc Welding Experiment

Considering shielded metal arc welding is the common method for the connection of steel structure construction by welding, the weld joint samples were prepared by shielded metal arc welding using a WS-300 DC welding power source (Ruiling Industry Co. Ltd., Shenzhen, China). A J857CrNi high-strength welding rod was chosen referring to the chemical composition and mechanical property of tested steels, and the chemical composition of the J857CrNi deposited metal is shown in Table 2. Considering the relatively high CE of tested steels, the weld preheating was conducted, and the tested steels were heated to 150 °C before welding. According to the welding quality and the common welding parameters used in real applications of tested steel, a unilateral 30° V shape groove was processed at the tested steel plate, and the welding current was 160 A. 

#### 2.2.2. Low-Cycle Fatigue Testing

**Figure 1:**
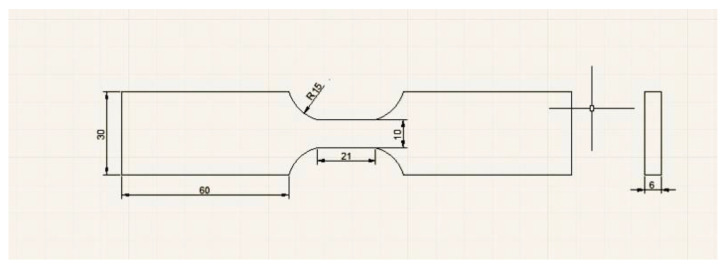
Size of low-cycle fatigue sample.

Low-cycle fatigue testing was carried out for the WJs by a Model 370.10 Landmark Servohydraulic Test Systems (MTS, Eden Prairie, USA) according to GB/T 15248-2008, and the size of the low-cycle fatigue sample is shown in Figure 1. Pull–push (axial) strain-controlled fatigue testing was performed in atmospheric condition at room temperature. A triangular waveform was selected with a frequency of 0.25 Hz and a strain ratio of R = −1. Different total strain amplitudes from 1.2% to 2.0% were applied, and the low-cycle fatigue property was measured thrice for every total strain amplitude. The low-cycle fatigue life (N_f_) was defined as the cycle number at which the fatigue sample fracture or the maximum stress decreased by 40%.

#### 2.2.3. Microstructural Characterization and Mechanical Testing

Metallographic samples of the BM and HAZ were prepared and polished; then, they were etched in 4% Nital solution for 15 s, and the microstructure was examined by a DSX510 optical microscope (OM) (Olymbus, Tokyo, Japan). Tensile samples of the WJs were prepared, and tensile testing was carried out by an Instron 5967 30 KN universal testing machine (Instron, High Wycombe, UK); the mechanical properties were measured thrice for the WJs of each steel.

The morphology of impact fracture and fatigue fracture of the HAZ was observed by a MERLIN Compact scanning electron microscope (SEM) (ZEISS, Oberkochen, Germany). Thin foils were cut from the BM of 10V8N steel, the HAZ of tested steels before and after LCF testing, and mechanically ground to 50–60 μm thick slices; then, the slices were twin jet electropolished with 5% perchloric acid alcohol solution at −30 °C and subsequently thinned by an ion beam. Thin foils were examined by a JEM 2100 transmission electron microscope (TEM) (JEOL, Tokyo, Japan) equipped with an EDS system (JEOL, Tokyo, Japan) to analyze the morphology of dislocation and precipitate and the elements of precipitate.

## 3. Results and Discussion

### 3.1. Microstructure, Mechanical Property and Impact Toughness

**Figure 2:**
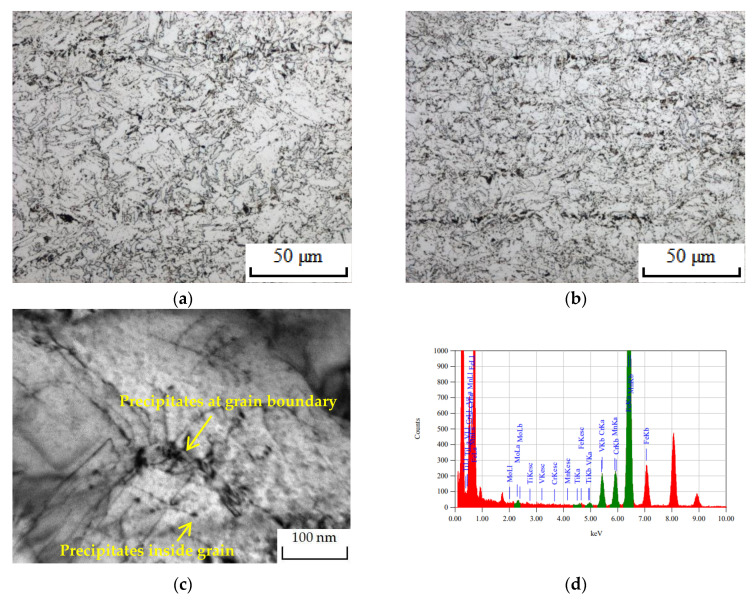
Microstructure of the tested steels: (**a**) metallographic structure of 0V steel: (**b**) metallographic structure of 10V8N steel; (**c**) precipitates inside grain and at grain boundary in 10V8N steel; (**d**) elemental analysis of precipitates by energy-dispersive spectroscopy (EDS).

The detailed microstructure of the tested steels is shown in Figure 2; the metallographic structure of both tested steels consisted of ferrite and pearlite. However, due to the nanoscale particles containing V precipitated at the grain boundary during hot rolling to give rise to the Zener pinning effect, the grain of 10V8N steel was relatively more refined; moreover, the nanoscale particles containing V also precipitated inside the grains, thereby contributing to grain refinement strengthening and precipitation strengthening. 

**Figure 3:**
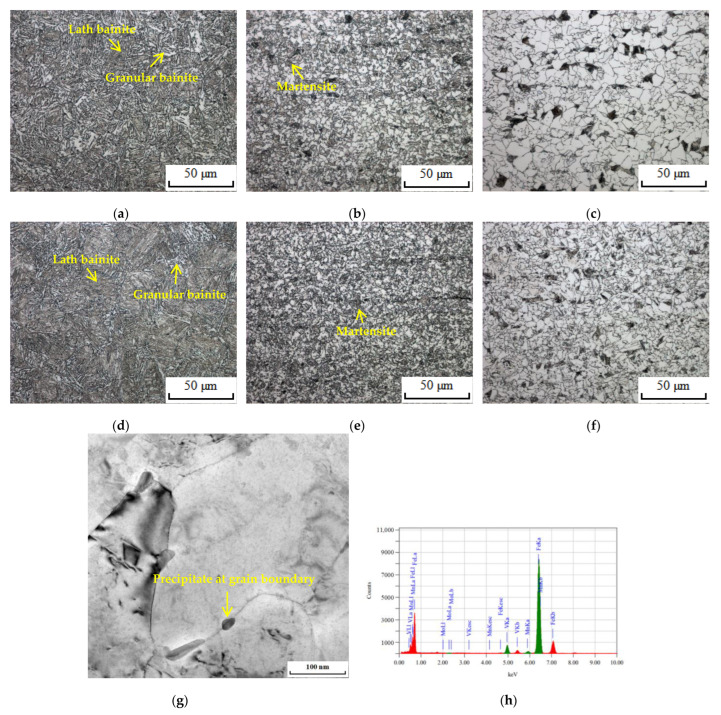
Metallographic structure of the HAZ and precipitates in the HAZ: (**a**) CGHAZ of 0V; (**b**) GRHAZ of 0V; (**c**) ICHAZ of 0V; (**d**) CGHAZ of 10V8N; (**e**) GRHAZ of 10V8N; (**f**) ICHAZ of 10V8N; (**g**) precipitate at grain boundary in the HAZ of 10V8N; (**h**) elemental analysis of the precipitate by energy dispersive spectroscopy (EDS).

The detailed microstructure of the HAZ of tested steels is shown in Figure 3. The metallographic structure of the coarse-grained heat-affected zone (CGHAZ) mainly consisted of lath bainite and granular bainite: specifically, the M–A constituent in an irregular block shape uniformly distributed in the ferrite grains to form granular bainite. The metallographic structure of the grain-refining heat-affected zone (GRHAZ) consisted of polygonal ferrite, pearlite, and martensite, and the metallographic structure of the intercritical heat-affected zone (ICHAZ) consisted of polygonal ferrite and pearlite, but comparing to 0V steel, the grains of GRHAZ and ICHAZ of 10V8N steel were relatively refined. It is considered that the dissolution and re-precipitation of the V element in the HAZ of 10V8N steel generated the nanoscale precipitates containing V to pin grain boundaries (Figure 3g,h), thereby leading to grain refinement.

**Figure 4:**
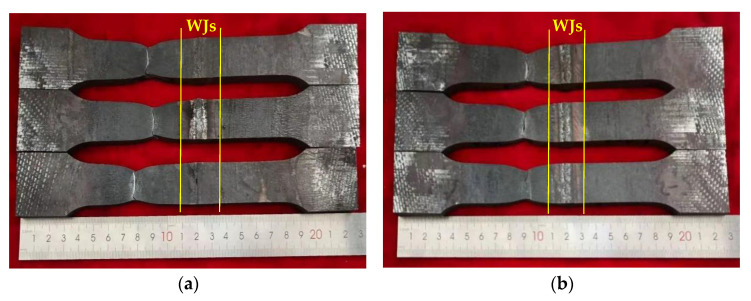
Macroscopic feature of tensile samples after tensile testing: (**a**) 0V; (**b**) 10V8N.

The macroscopic feature of typical tensile samples after tensile testing is shown in Figure 4, and the mechanical properties of the WJs are shown in Table 3. The fracture position of tensile samples was located at the BM, indicating that the welding parameters and welding material matched well, and the strength of the WJs was higher. The strength of 10V8N steel was higher; meanwhile, the yield ratio of 10V8N steel was 0.81, which is considered to increase the seismic resistance of steel structure construction. 

**Figure 5:**
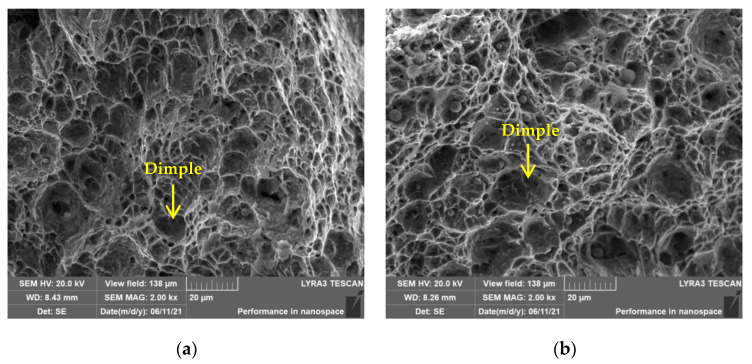
Morphology of impact fracture of the HAZ: (**a**) 0V; (**b**) 10V8N.

The impact values at −20 °C of the HAZ of tested steels are shown in Table 4. The impact toughness of the HAZ of tested steels were good, and the impact values of the HAZ of 10V8N steel were only slightly lower than that of the HAZ of 0V steel. The typical impact fracture morphology of the HAZ is shown in Figure 5, and the dimples were observed. Micro-holes caused by plastic deformation experienced nucleation, growth, and aggregation to generate dimples. The impact fracture of the HAZ of tested steels was basically composed of dimples, and the dimples were large and deep, indicating that the impact fracture of the HAZ of tested steels was characterized by ductile fracture. Therefore, it was further proved that the welding parameters and welding material matched well.

### 3.2. Low-Cycle Fatigue Life

**Figure 6:**
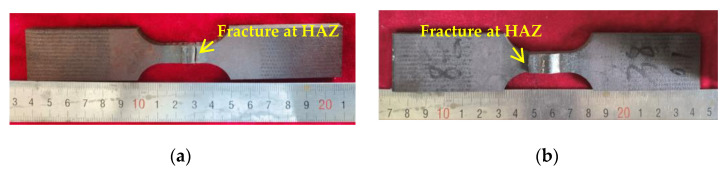
Macroscopic feature of fatigue samples after LCF testing: (**a**) 0V; (**b**) 10V8N.

The macroscopic feature of typical fatigue samples after LCF testing is shown in Figure 6. All fatigue samples fractured at the HAZ, indicating the HAZ was a weak zone under the LCF condition, and the seismic resistance of steel structure construction welded by the tested steels greatly depends on the LCF property of the HAZ.

The N_f_ values of the WJs under various total strain amplitudes are shown in Table 5. When the total train amplitude (Δε/2) was 1.6% or less, the N_f_ values of the WJs of 10V8N steel were significantly longer. Specifically, when the Δε/2 was 1.2%, the N_f_ of the WJ of 10V8N steel was about 3.5 times that of the WJ of 0V, when the Δε/2 values were 1.4% and 1.6%, the N_f_ values of the WJs of 10V8N steel were about twice that of the WJs of 0V steel. However, when the Δε/2 increased to 1.8% and 2.0%, the N_f_ values of the WJs of both tested steels were close, which reduced to less than 600 cycles, and the N_f_ values of the WJs of 10V8N steel were slightly shorter than those of the WJs of 0V steel.

### 3.3. Cyclic Stress Response Curves

**Figure 7:**
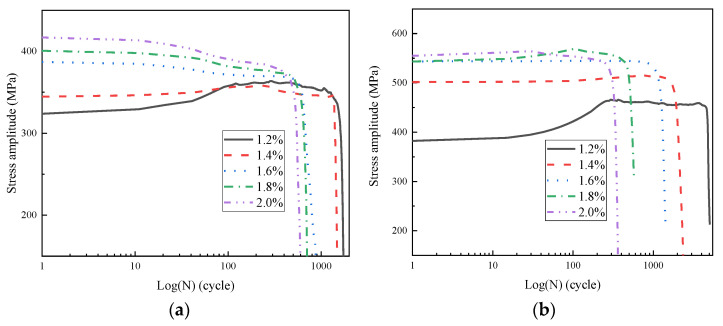
Cyclic stress response curves of the WJs: (**a**) 0V; (**b**) 10V8N.

The cyclic stress response curve is the evolution curve of the stress amplitude response with the cyclic cycle under various Δε/2, which represents the stress variation characteristic that the material can bear in a certain cyclic cycle under alternation load, and it is an important LCF property characteristic. The typical cyclic stress response curves of the WJs are shown in Figure 7. The cyclic stress response characteristic of the material under the strain-controlled LCF condition can be manifested as cyclic hardening, cyclic stabilization and cyclic softening. With the increase in the cyclic cycle, the increasing stress amplitude means that the material experiences cyclic hardening, and the stable stress amplitude means that the material experiences cyclic stabilization, while the decreasing stress amplitude means that the material experiences cyclic softening. For the WJs of 0V steel, when the Δε/2 values were 1.2% and 1.4%, they experienced cyclic hardening in the initial stage, and then, they experienced cyclic stabilization when the Δε/2 was 1.2% or cyclic softening when the Δε/2 was 1.4% until failure; when the Δε/2 values were 1.6%, 1.8% and 2.0%, they experienced cyclic softening in the initial stage and then experienced short cyclic stabilization when the Δε/2 was 1.6% or had ongoing cyclic softening when the Δε/2 values were 1.8% and 2.0% until failure. For the WJs of 10V8N steel, when the Δε/2 values were 1.2% and 1.4%, they experienced cyclic hardening in the initial stage and then experienced cyclic stabilization until failure; when the Δε/2 was 1.6%, it kept cyclic stabilization until failure; when the Δε/2 values were 1.8% and 2.0%, they experienced cyclic hardening in the initial stage and then experienced short cyclic softening until failure. When the material is subjected to cyclic softening under cyclic load, the strength of the material will decrease to result in premature failure, which is not conductive to improving the dynamic bearing capacity [23,29]. Compared with the WJs of 0V steel, the cyclic-softening behavior of the WJs of 10V8N steel was improved when the Δε/2 ≥ 1.4%. Moreover, under the LCF condition, fatigue damage accumulated rapidly when the Δε/2 ≥ 1.8%, and fatigue cracks generated and propagated in fewer cyclic cycles, leading to the decrease in the bearing capacity of the material to experience cyclic softening and failure rapidly. 

It is generally recognized that the cyclic stress response characteristic of the material can be explained by the movement and interaction of dislocations under cyclic load [23,30,31]. During the fatigue process, the dislocation proliferation and slip in the material take place constantly under cyclic load. On the one hand, the dislocation density decreases with dynamic recovery through the rearrangement of the dislocation grid to a lower energy state; on the other hand, the dislocation density increases through dislocation proliferation and entanglement to inhibit dislocation movement. When the dynamic recovery of the dislocation dominates, the deformation resistance of the material will decrease; thereby, the material will experience cyclic softening; when the dynamic recovery, proliferation and entanglement of the dislocation reach a dynamic equilibrium, the deformation resistance of the material will remain stable; thereby, the material will experience cyclic stabilization; when the proliferation and entanglement of the dislocation dominate, the deformation resistance of the material will increase; thereby, the material will experience cyclic hardening.

**Figure 8:**
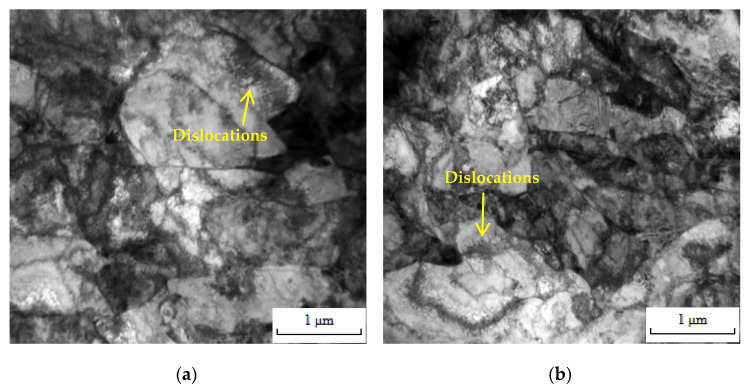

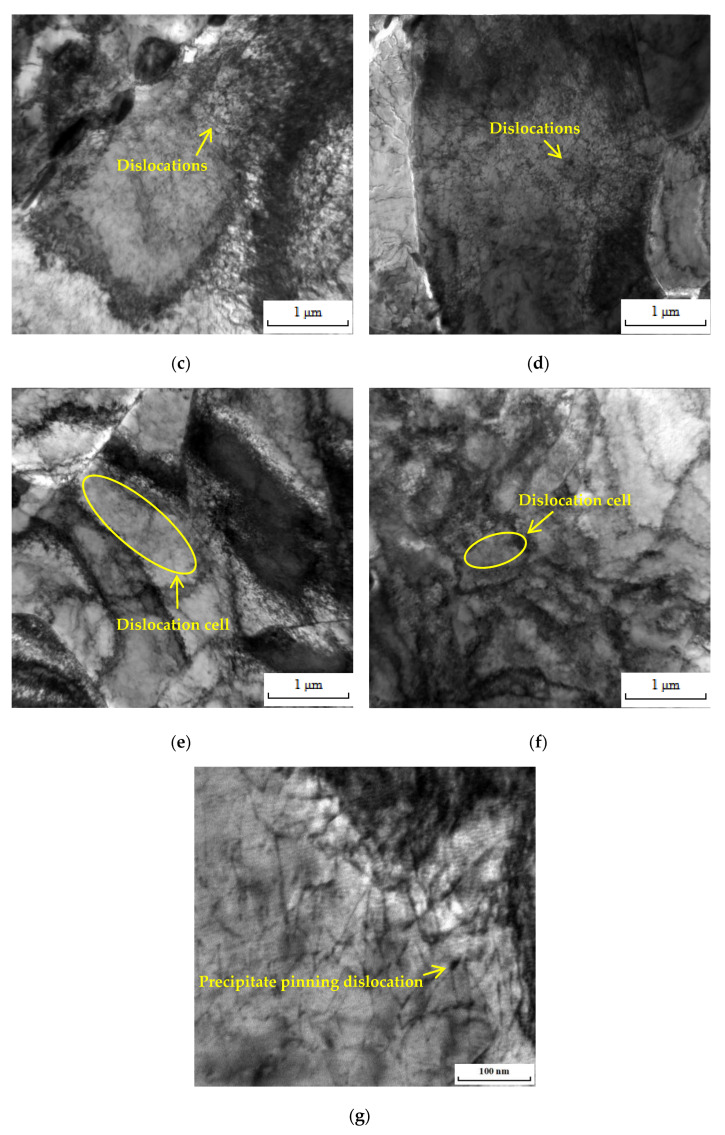
Dislocations in the HAZ of tested steels before and after LCF testing under 1.8% strain amplitude: (**a**) dislocations in the HAZ of 0V steel before LCF testing; (**b**) dislocations in the HAZ of 10V8N steel before LCF testing; (**c**) dislocations in the HAZ of 0V steel after LCF testing; (**d**) dislocations in the HAZ of 10V8N steel after LCF testing; (**e**) dislocation cells in the HAZ of 0V steel after LCF testing; (**f**) dislocation cells in the HAZ of 10V8N steel after LCF testing; (**g**) precipitates pinning the dislocations in the HAZ of 10V8N steel.

The typical morphology of dislocation in the HAZ of tested steels before and after LCF testing under 1.8% Δε/2 is shown in Figure 8. The dislocation density in the HAZ of tested steels before LCF testing was close (Figure 8a,b); however, compared with the HAZ of 0V steel, the dislocation density in the HAZ of 10V8N steel after LCF testing was higher (Figure 8c,d), indicating that the dislocation proliferation during LCF testing was more obvious. Moreover, under cyclic load, the dislocation entanglement formed dislocation walls, and the dislocation walls were enclosed into an independent, dislocation-free area to form a dislocation cell. Compared with the HAZ of 0V steel, the dislocation cells in the HAZ of 10V8N steel after LCF testing were smaller and more frequent (Figure 8e,f), indicating that the dislocation entanglement was more serious during LCF testing to inhibit the dislocation movement. The dissolution and re-precipitation of the V element in the HAZ of 10V8N steel generated nanoscale precipitates containing V to pin grain boundaries, thereby leading to grain refinement to increase the grain boundary area (Figure 3), which is conductive to inhibiting dislocation movement. Meanwhile, the nanoscale precipitates can pin the dislocation and promote dislocation pile-up (Figure 8g), thereby enhancing the dislocation proliferation and entanglement during LCF testing [23,32,33]. Therefore, when the Δε/2 was 1.8%, the WJs of 0V steel experienced cyclic softening, while the WJs of 10V8N steel experienced cyclic hardening due to the significant dislocation proliferation and entanglement in the HAZ to increase the deformation resistance.

### 3.4. Hysteresis Loops

**Figure 9:**
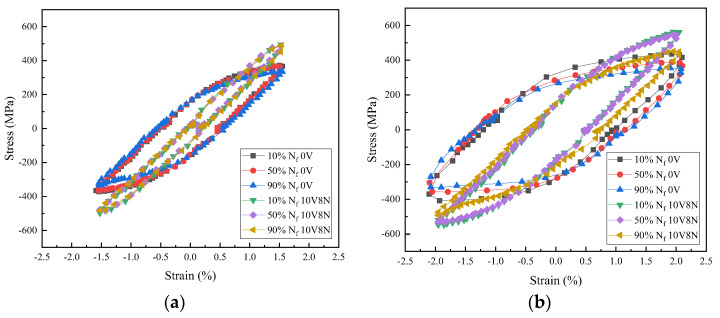
Hysteresis loops of the WJs: (**a**) under 1.6% Δε/2; (**b**) under 2.0% Δε/2.

The hysteresis loop reflects the stress–strain response under cyclic load, the typical hysteresis loops of the WJs when the Δε/2 were 1.6% and 2.0% are shown in Figure 9. The hysteresis loops are continuous, smooth and symmetrical, indicating that the process of the LCF testing was well controlled. 

The area covered by the hysteresis loops of each cycle represents the energy consumed in the cycle, most of which is transformed into irreversible plastic strain energy. The more plastic strain energy the material accumulates, the greater its plastic deformation, and the faster material damage occurs [33,34,35]. With the increase in Δε/2, the area covered by the hysteresis loops increased, indicating the plastic strain energy absorbed by the WJs increased; thereby, the material damage was faster, leading to the reduction in N_f_. From the cyclic initial stage (10% N_f_) to the cyclic stable stage (50% N_f_), and then to the cyclic later stage (90% N_f_), the area covered by the hysteresis loops gradually decreased, indicating that with the increase in the cyclic cycles, the damage accumulation resulted in the decrease in the energy absorption capacity of the WJs.

The Δε/2 of LCF testing consists of elastic strain amplitude (Δε_e_/2) and plastic strain amplitude (Δε_p_/2), and the width of the hysteresis loop is the Δε_p_ when the stress is 0 [35]. The typical Δε_e_/2 and Δε_p_/2 values of the WJs at the cyclic stable stage (50% N_f_) are shown in Table 6. Comparing the hysteresis loops of the WJs under the same Δε/2, the area covered by the hysteresis loops of the WJs of 10V8N steel were smaller, which is mainly due to the smaller Δε_p_/2, indicating that the WJs of 10V8N steel mainly took place elastic deformation under cyclic load; thereby, the absorbed plastic strain energy was less to reduce the material damage accumulation, which was conductive to improving the N_f_. Therefore, when the Δε/2 ≤ 1.6%, the N_f_ of the WJs of 10V8N steel was significantly longer than that of the WJs of 0V steel. However, when the Δε/2 increased to 1.8% and 2.0%, the N_f_ of the WJs of both tested steels decreased to less than 600 cycles, and the N_f_ values of the WJs of 10V8N steel decreased faster, which were even slightly shorter than those of the WJs of 0V steel. In order to explain this phenomenon, it is necessary to investigate the plastic strain energy density (ΔW_p_).

**Figure 10:**
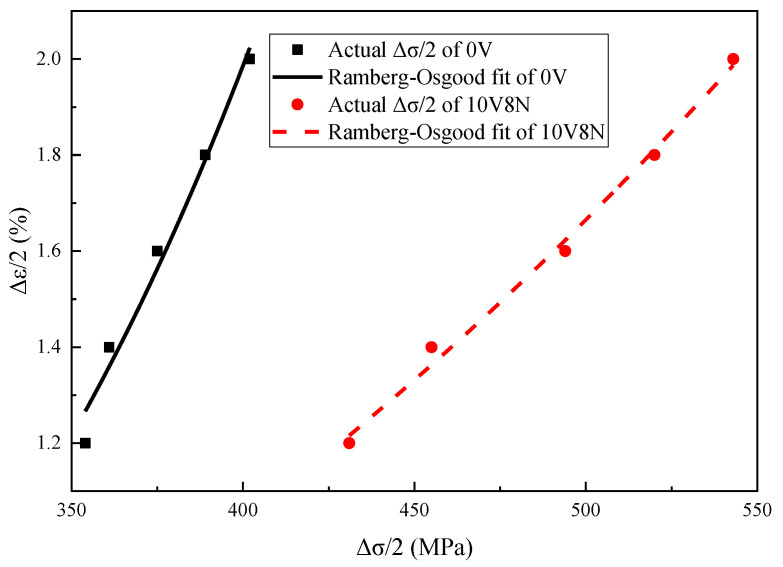
Cyclic stress–strain relationship and the Ramberg–Osgood fits of the WJs.

The cyclic stress–strain curve can be obtained by connecting the peaks of the stable hysteresis loops under various Δε/2; it is applied to represent the stable stress–strain relationship of the material under cyclic load, and it is usually described by a Ramberg–Osgood equation (Equation (2)) [16,33,36,37]. The typical total stress amplitude (Δσ/2) of the WJs under various Δε/2 at the cyclic stable stage is shown in Table 6, and the Young’s modulus (E) is shown in Table 3. According to Equation (2), the cyclic stress–strain relationship of the WJs of 0V steel and 10V8N steel can be obtained as shown in Equation (3) and Equation (4), respectively, and the fitting effect is good, according to Figure 10.

(2) $$\frac{\Delta \varepsilon }{2}=\frac{{\Delta \varepsilon }_{e}}{2}+\frac{{\Delta \varepsilon }_{p}}{2}=\frac{\Delta \sigma }{2E}+{\left(\frac{\Delta \sigma }{2K^{\prime }}\right)}^{\frac{1}{n^{\prime }}}$$

where Δε, Δε_e_, Δε_p_, E, Δσ, K′, and n′ are the total strain range, total elastic strain range, total plastic strain range, Young’s modulus, cyclic-hardening coefficient, and cyclic-hardening exponent, respectively.

(3) $$\frac{\Delta \varepsilon }{2}=\frac{\Delta \sigma }{2\times 206,529}+{\left(\frac{\Delta \sigma }{2\times 1084.027}\right)}^{\frac{1}{0.248}}$$

(4) $$\frac{\Delta \varepsilon }{2}=\frac{\Delta \sigma }{2\times 214,316}+{\left(\frac{\Delta \sigma }{2\times 3123.837}\right)}^{\frac{1}{0.432}}$$

**Figure 11:**
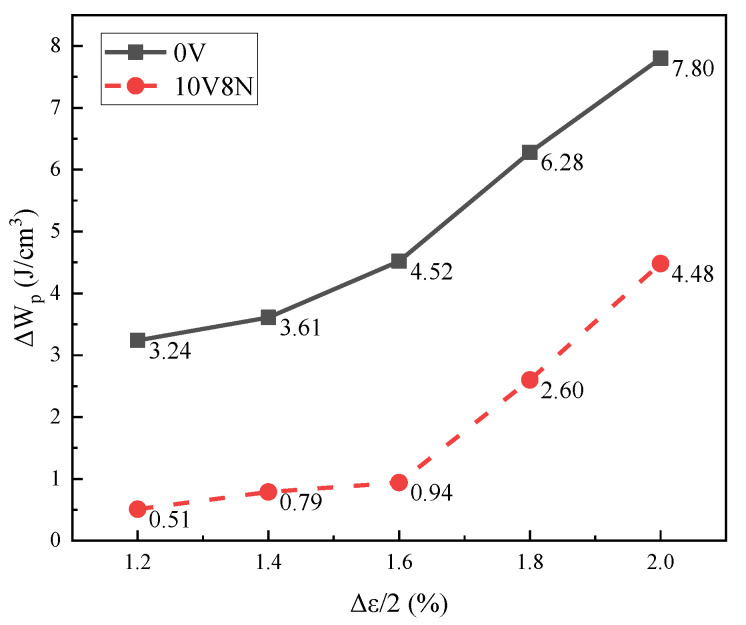
Plastic strain energy density (ΔW_p_) of the WJs at cyclic stable stage (50% N_f_).

The plastic strain energy density (ΔW_p_) of each cycle can be calculated by the Halford–Morrow equation (Equation (5)) [12,38]. According to the values of Δσ and Δε_p_ in Table 6 and the values of n′ in Equations (3) and (4), the values of ΔW_p_ of the WJs at the cyclic stable stage can be calculated, and the results are shown in Figure 11. When the Δε/2 > 1.6%, the growth rate of ΔW_p_ of the WJs increased with the increase in Δε/2. Especially for the WJs of 10V8N steel, the Δε_p_/2 increased significantly with the increase in Δε/2, leading to a greater change in the growth rate of ΔW_p_; thereby, the material damage accumulation was faster. Moreover, according to the microstructure analysis (Figure 3 and Figure 7), when the Δε/2 was large, the nanoscale precipitates containing V in the HAZ of 10V8N steel played a role in grain refinement and dislocation pinning to enhance dislocation proliferation and entanglement, thereby causing stress concentration to make the material more easily reach the strength limit. Therefore, with the increase in Δε/2 to 1.8% and 2.0%, the N_f_ values of the WJs of 10V8N steel decreased significantly to become slightly shorter than those of the WJs of 0V steel.

(5) $$\Delta W_{p}=\left(\frac{1-n^{\prime }}{1+n^{\prime }}\right)\Delta \sigma \Delta \varepsilon_{p}$$

### 3.5. Fatigue Fracture Morphology

**Figure 12:**
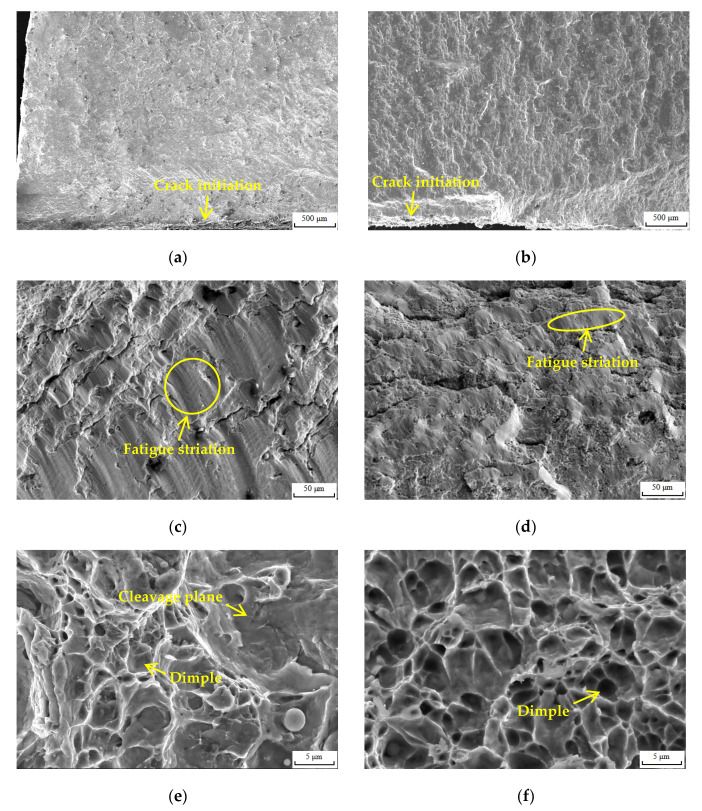
Morphology of fatigue fractures under 1.6% total strain amplitude: (**a**) crack initiation region of 0V WJ; (**b**) crack initiation region of 10V8N WJ; (**c**) crack propagation region of 0V WJ; (**d**) crack propagation region of 10V8N WJ; (**e**) ultimate fracture region of 0V WJ; (**f**) ultimate fracture region of 10V8N WJ.

The typical fatigue fracture morphology when the Δε/2 was 1.6% is shown in Figure 12, and the fatigue fracture consisted of a crack initiation region, crack propagation region and ultimate fracture region. The morphology of crack initiation region is shown in Figure 12a,b. The crack initiation on the surface of the samples was mainly due to the fact that the surface of the samples was in the plane stress state. Thereby, the plastic slip easily occurred and formed slip bands, which was conductive to the nucleation of micro-cracks [34]. The morphology of the crack propagation region is shown in Figure 12c,d; obvious fatigue striations, micro-holes and secondary cracks were observed. In each cycle, when the sample was subjected to compressive stress amplitude, the fatigue crack was inhibited and gradually closed; when the sample was subjected to tensile stress amplitude, the fatigue crack opened and propagated gradually, and the propagation direction was perpendicular to the fatigue striation. Therefore, the length that the fatigue crack can propagate in one cycle was the size of the fatigue striation [34]. When the Δε/2 was 1.6%, the fatigue striation in the crack propagation region of the WJ of 10V8N steel was small, indicating that the fatigue crack propagation was inhibited. Moreover, there were more micro-holes and secondary cracks in the crack propagation region of the WJ of 10V8N steel, which reduced the degree of stress concentration and further relieved the propagation of the main crack [39]. The morphology of the ultimate fracture region is shown in Figure 12e,f, and dimples were observed. When the Δε/2 was 1.6%, the dimples in the ultimate fracture region of the WJ of 0V steel were shallower, and some cleavage planes were observed, while the deep dimples were observed in the ultimate fracture region of the WJ of 10V8N steel, indicating that the crack resistance of the WJ of 10V8N steel was greater.

According to the analysis of fatigue fracture morphology, the ability to inhibit the fatigue crack propagation of the WJ of 10V8N steel was stronger under 1.6% Δε/2; it is considered that the greater crack resistance was mainly due to the re-precipitation of the nanoscale precipitates containing V in the HAZ of 10V8N steel. The nanoscale precipitates refined the grains to increase the area of the grain boundary, which was conductive to inhibiting crack propagation. Meanwhile, the main crack propagation was inhibited when it met the nanoscale precipitates, and micro-holes and secondary cracks were formed there to consume the energy of the main crack propagation.

### 3.6. Low-Cycle Fatigue Life Prediction

**Figure 13:**
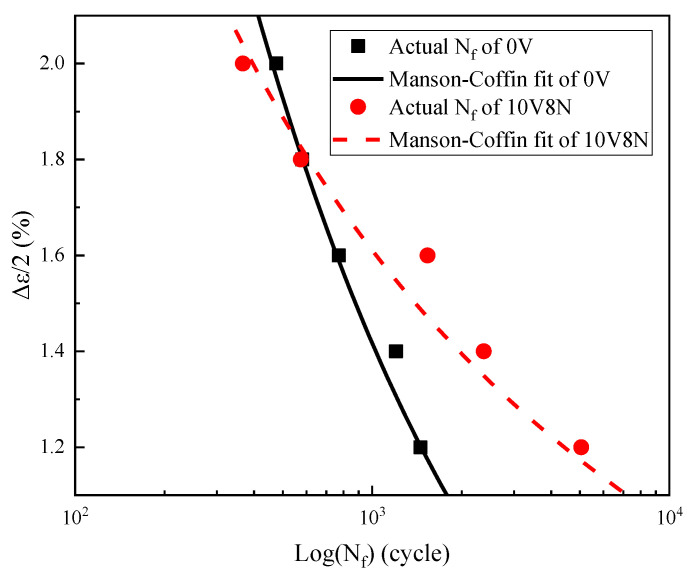
Prediction of low-cycle fatigue life (N_f_) of the WJs based on Manson–Coffin equation.

The N_f_ of the WJs can be predicted with the Manson–Coffin equation (Equation (6)), which is generally applied to the LCF with the cyclic cycles less than 10^4^ [17,33,40,41,42], and Equations (7) and (8) can be obtained through a derivation of Equation (6). Based on the measured N_f_ (Table 5), Δε_e_/2 and Δε_p_/2 (Table 6) of the WJs under various Δε/2, as well as the Young’s modulus (E) is shown in Table 3., the values of σ_f_′, b, ε_f_′ and c can be obtained by fitting Equations (7) and (8), thereby obtaining the N_f_ prediction equations of the WJs of 0V steel and 10V8N steel shown as Equations (9) and (10), respectively. According to Figure 13, the fitting effect is good, and the N_f_ of the WJs of 10V8N steel is longer than that of the WJs of 0V steel when the Δε/2 < 1.8%, while the N_f_ of the WJs of 10V8N steel is gradually shorter than that of the WJs of 0V steel when the Δε/2 ≥ 1.8%.

(6) $$\frac{\Delta \varepsilon }{2}=\frac{\Delta \varepsilon_{e}}{2}+\frac{\Delta \varepsilon_{p}}{2}=\frac{{\sigma_{f}}^{\prime }}{E}{\left(2N_{f}\right)}^{b}+{\varepsilon_{f}}^{\prime }{\left(2N_{f}\right)}^{c}$$

where σ_f_′, b, ε_f_′, and c are the fatigue strength coefficient, fatigue strength exponent, fatigue ductility coefficient, fatigue ductility exponent, respectively.

(7) $$\mathrm{lg}(\frac{\Delta \varepsilon_{e}}{2})=\mathrm{lg}(\frac{{\sigma_{f}}^{\prime }}{E})+b\mathrm{lg}(2N_{f})$$

(8) $$\mathrm{lg}(\frac{\Delta \varepsilon_{p}}{2})=\mathrm{lg}({\varepsilon_{f}}^{\prime })+c\mathrm{lg}(2N_{f})$$

(9) $$\frac{\Delta \varepsilon }{2}=0.158\times {\left(2N_{f}\right)}^{0.369}+0.405\times {\left(2N_{f}\right)}^{0.589}$$

(10) $$\frac{\Delta \varepsilon }{2}=0.043\times {\left(2N_{f}\right)}^{0.147}+0.355\times {\left(2N_{f}\right)}^{0.671}$$

### 3.7. Cyclic Toughness

**Figure 14:**
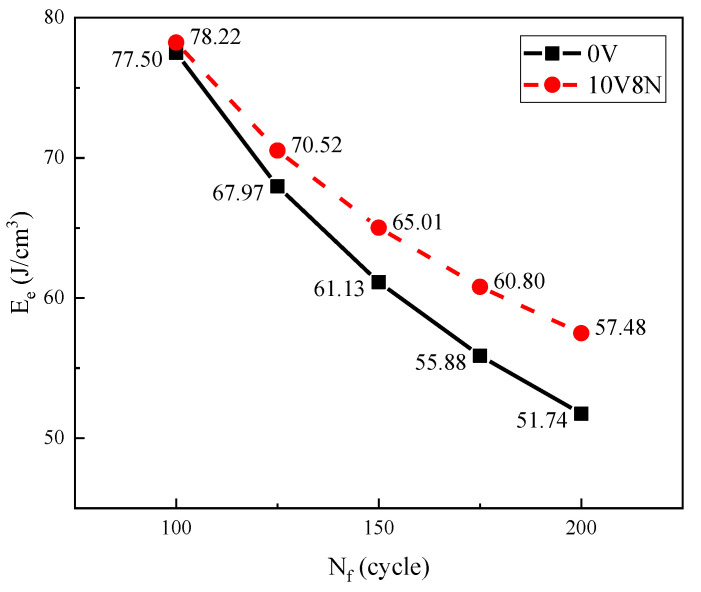
Cyclic toughness (E_e_) values of the WJs when the low-cycle fatigue life (N_f_) is between 100 and 200 cycles.

The damage to the steel structure caused by earthquakes depends on the seismic energy input and the energy absorbed by the steel structure; the steel structure is usually damaged in 100 to 200 cycles of seismic waves under strong earthquake. Therefore, the greater the energy absorbed by the material of the steel structure in 100 to 200 cycles, the better its seismic resistance. The product of Δσ and Δε under the LCF condition is defined as cyclic toughness (E_e_) (Equation (11)), which represents the energy absorption rate of the material under cyclic load. Therefore, when N_f_ is between 100 and 200 cycles, the larger the E_e_ value, the higher the seismic energy that the material can absorb and the better the seismic resistance of the material [6,43,44,45]. In the condition of LCF, when the N_f_ of the WJs is between 100 and 200 cycles, the Δε can be obtained by Equations (9) and (10); then, the Δσ can be obtained by Equations (3) and (4), and the values of E_e_ can be obtained by Equation (11) eventually; the specific results are shown in Figure 14. When the N_f_ is between 100 and 200 cycles, the E_e_ value of the WJ of 10V8N steel is larger than that of the WJ of 0V steel, indicating that it is able to absorb more energy in seismic conditions; therefore, the seismic resistance of the WJ of 10V8N steel is better.

(11) $$E_{e}=\Delta \sigma \Delta \varepsilon$$

## 4. Conclusions

LCF testing is carried out for the WJs of 0V and 10V8N constructional steels to investigate the effects of V-N microalloying on the LCF property and the seismic resistance of the WJs of constructional steel, which led to the following conclusions:

1. According to the measured results and the prediction results of N_f_, the N_f_ values of the WJs of 10V8N steel were significantly longer than those of the WJs of 0V steel when Δε/2 ≤ 1.6%, while the N_f_ values of the WJs of 10V8N steel were gradually shorter than those of the WJs of 0V steel when Δε/2 > 1.6%.
2. Under the same Δε/2, compared with the WJs of 0V steel, the Δε_p_/2 of the WJs of 10V8N steel was smaller; thereby, there was less plastic strain energy absorbed that could reduce the material damage accumulation, which was conductive to improving the N_f_. Therefore, when Δε/2 ≤ 1.6%, the N_f_ of the WJs of 10V8N steel was significantly longer than that of the WJs of 0V steel. However, when Δε/2 > 1.6%, with the increase in Δε/2, compared with the WJs of 0V steel, the ΔW_p_ growth rate of the WJs of 10V8N steel increased more significantly, leading to faster material damage accumulation. Meanwhile, the nanoscale precipitates containing V in the HAZ of 10V8N steel played a role in grain refinement and dislocation pinning to enhance dislocation proliferation and entanglement, thereby causing the stress concentration to make the material more easily reach the strength limit. Therefore, when Δε/2 > 1.6%, the N_f_ of the WJs of 10V8N steel decreased significantly to slightly shorter than that of the WJs of 0V steel.
3. When the Δε/2 is 1.6%, compared with the WJ of 0V steel, the nanoscale precipitates containing V in the HAZ of 10V8N steel refined the grains and inhibited the main crack propagation, leading to smaller fatigue striation in the crack propagation region, more micro-holes and secondary cracks in the crack propagation region, and the deep dimples in the ultimate fracture region; therefore, the crack resistance of the WJ of 10V8N steel was greater.
4. When the N_f_ is between 100 and 200 cycles, the E_e_ value of the WJ of 10V8N steel is larger than that of the WJ of 0V steel, indicating that it is able to absorb more energy in seismic conditions; therefore, the seismic resistance of the WJ of 10V8N steel is better.

## Figures and Tables

**Table 1:** Chemical composition and carbon equivalent values of the tested steels (wt %).

Steel	C	Si	Mn	P	S	Cu	Ni	Cr	V	Als	N	CE
0V	0.07	0.39	1.45	0.011	0.002	0.36	0.31	0.63	-	0.036	0.0021	0.48
10V8N	0.07	0.38	1.47	0.008	0.003	0.34	0.31	0.62	0.10	0.040	0.0078	0.50

**Table 2:** Chemical composition of J857CrNi deposited metal (wt %).

Elements	C	Si	Mn	Ni	Cr	Mo
Reference content	≤0.10	≤0.80	≥0.50	≥1.75	≥0.30	≥0.20

**Table 3:** Mechanical property of the WJs.

Steel	Yield Strength R_eL_ (MPa)	Tensile Strength R_m_ (MPa)	Yield Ratio R_eL_/R_m_	Elongation A (%)	Young’s Modulus E (MPa)	Fracture Position
0V	326 ± 7	473 ± 10	0.69 ± 0	32.5 ± 1.5	206,529 ± 3099	BM
10V8N	488 ± 10	600 ± 11	0.81 ± 0.01	23.5 ± 1.2	214,316 ± 4196	BM

**Table 4:** Low-temperature impact value of the HAZ.

Steel	−20 °C Impact Value (J)			
	Value 1	Value 2	Value 3	Mean Value
0V	101	105	109	105
10V8N	83	98	101	94

**Table 5:** Low-cycle fatigue life (N_f_) of the WJs under various total strain amplitudes (cycle).

Δε/2 (%)	0V	10V8N
1.2	1452 ± 87	5050 ± 129
1.4	1202 ± 82	2372 ± 100
1.6	772 ± 29	1535 ± 80
1.8	581 ± 41	575 ± 38
2.0	389 ± 22	367 ± 29

**Table 6:** Elastic strain amplitude (Δε_e_/2), plastic strain amplitude (Δε_p_/2) and total stress amplitude (Δσ/2) of the welded joints at cyclic stable stage (50% N_f_).

Δε/2 (%)	Δε_e_/2 (%)		Δε_p_/2 (%)		Δσ/2 (MPa)	
	0V	10V8N	0V	10V8N	0V	10V8N
1.2	0.44	1.05	0.76	0.15	354	431
1.4	0.57	1.18	0.83	0.22	361	455
1.6	0.60	1.36	1.00	0.24	375	494
1.8	0.46	1.17	1.34	0.63	389	520
2.0	0.39	0.96	1.61	1.04	402	543

## Data Availability

The study did not report any data.
